# Lean Yet Unhealthy: Asian American Adults Had Higher Risks for Metabolic Syndrome than Non-Hispanic White Adults with the Same Body Mass Index: Evidence from NHANES 2011–2016

**DOI:** 10.3390/healthcare9111518

**Published:** 2021-11-08

**Authors:** Lin Zhu, Wei J. Yang, Cody B. Spence, Aisha Bhimla, Grace X. Ma

**Affiliations:** 1 Center for Asian Health, Lewis Katz School of Medicine, Temple University, 3440 N. Broad St., Philadelphia, PA 19140, USA; jennyweiyang@temple.edu (W.J.Y.); aisha.bhimla@temple.edu (A.B.); grace.ma@temple.edu (G.X.M.); 2 Department of Sociology, College of Liberal Arts, Temple University, 1114 W. Berks St., Philadelphia, PA 19122, USA; cspence@temple.edu; 3 Department of Clinical Sciences, Lewis Katz School of Medicine, Temple University, 3440 N. Broad St., Philadelphia, PA 19140, USA

**Keywords:** metabolic syndrome, body mass index, Asian American, racial differences

## Abstract

(1) Background: Despite having consistently lower rates of obesity than other ethnic groups, Asian Americans (AAs) are more likely to be identified as metabolically obese, suggesting an ethnic-specific association between BMI and cardiometabolic outcomes. The goal of this study was to provide an estimate of metabolic syndrome (MetS) prevalence among AAs using national survey data and to compare this rate to that of non-Hispanic Whites (NHWs) over the BMI continuum. (2) Methods: Using the NHANES 2011–2016 data, we computed age-adjusted, gender-specific prevalence of MetS and its individual components for three BMI categories. Furthermore, we conducted multivariate binary logistic regression to examine the risk of MetS in AAs compared to NHWs, controlling for sociodemographic and lifestyle factors. The analysis sample consisted of 2121 AAs and 6318 NHWs. (3) Results: Among AAs, the prevalence of MetS and its components increased with higher BMI levels, with overall prevalence being 5.23% for BMI < 23, 38.23% for BMI of 23–27.4, and 77.68% for BMI ≥ 27.5 in men; and 18.61% for BMI < 23, 47.82% for BMI of 23–27.4, and 67.73% for BMI ≥ 27.5 in women. We also found that for those with a BMI > 23, AAs had a higher predicted risk of MetS than their NHW counterparts of the same BMI level, in both men and women. (4) Conclusions: Our findings support the use of lower BMI ranges for defining overweight and obesity in Asian populations, which would allow for earlier and more appropriate screening for MetS and may better facilitate prevention efforts.

## 1. Introduction

Metabolic syndrome (MetS) is a collection of metabolic conditions associated with an increased risk of type 2 diabetes mellitus (T2DM), cardiovascular disease (CVD), and all-cause mortality [1,2,3,4,5,6]. Its main components are abdominal obesity, insulin resistance, high blood pressure, and dyslipidemia [7,8,9,10]. In recent years, MetS has reached epidemic proportions globally [3], affecting approximately one-quarter of the world’s population [6,11]. Therefore, there has been increased attention from researchers and healthcare providers toward MetS and its components as important treatment targets for reducing cardiovascular disease and related conditions.

Defined by specific body habitus, lipid metabolism, and insulin sensitivity [12,13,14,15,16], the Asian American population affords a valuable model to disentangle the roles of biological, sociocultural, and behavioral factors in cardiometabolic etiology [17,18,19]. Researchers have found that despite having consistently lower body mass index (BMI) than other ethnic groups [20,21,22], Asian Americans in aggregate and certain subgroups are at higher risk for hypertension [23,24,25], elevated blood glucose [26,27,28], and CVD [29,30,31]. Global data indicate that individuals of Asian descent are more likely than non-Hispanic Whites to be identified as metabolically obese but normal weight (MONW) [32,33]. Compared to individuals with similar BMI levels, subjects identified as MONW are often characterized by having altered insulin sensitivity and lipid profile, greater abdominal adiposity, and higher blood pressure [34]. The “lean yet unhealthy” phenomenon in Asian Americans is thought to be the result of greater percentage of body fat accumulation at a given BMI level [32], suggesting an ethnic-specific association between body fat percentage and BMI [13].

The association between MetS and BMI among Asian Americans is understudied [29,35,36]. One study used data from the electronic health records of primary care patients in the San Francisco Bay Area and found that at each level of BMI, Asian Americans were more likely to manifest MetS than non-Hispanic Whites [20]. While the use of data from a single geographic area limited the generalizability of the results, the study adds to the cumulative evidence that population-specific BMI ranges are necessary for accurate, timely diagnosis of overweight and obesity and for cardiometabolic risk prediction. A comprehensive examination of MetS prevalence in Asian Americans is crucial in estimating the current and future burden of CVD [37].

In the present study, the first goal was to provide estimates of the prevalence rates of MetS and its components among a nationally representative sample of Asian Americans. The second goal was to examine the risk of MetS by BMI level in Asian Americans in comparison to non-Hispanic Whites. We determined the presence of MetS in aggregated samples and in the five largest Asian subgroups in the United States—Chinese, Filipino, Asian Indian, Vietnamese, and Korean. With the marked heterogeneity in cardiometabolic risks and social/behavioral factors found in Asian Americans [38], we hypothesized that there would be significant differences in the risk of MetS at each level of BMI across demographic groups.

## 2. Materials and Methods

### 2.1. Study Sample

This study is a cross-sectional study of non-Hispanic Asian (hereafter referred to as Asian) adults from the National Health and Nutrition Examination Survey (NHANES) 2011–2016. We combined the data from three release cycles: 2011–2012 [39], 2013–2014 [40], and 2015–2016 [41]. NHANES is one of a series of health-related programs conducted by the Centers for Disease Control and Prevention’s (CDC) National Center for Health Statistics (NCHS). The objective of NHANES is to monitor trends in the prevalence of selected diseases and to study the relationship between diet, nutrition, and health [39,40,41]. NHANES uses a multistage, stratified design to produce a study sample that is representative of the noninstitutionalized civilian resident population in the 50 states and the District of Columbia. Every year, approximately 5000 individuals of all ages are interviewed in their homes and complete the health examination component of the survey [39,40,41]. The survey included two parts. First, survey questionnaires were administered to eligible participants at home, where person-level demographics, health, and nutrition information were collected. Then, participants were invited to visit specially equipped mobile examination centers (MECs) for a standardized health examination. The survey procedures are detailed elsewhere [42]. The 2011–2016 NHANES oversampled Asian and several other subpopulations to increase the precision of estimates for these groups. To facilitate the oversampling of the Asian population, NHANES provided survey materials and a promotional video in traditional and simplified Mandarin, Korean, and Vietnamese [39,40,41].

### 2.2. Measures

Metabolic syndrome. To define MetS, we used the 2005 definition from the International Diabetes Federation (IDF) [43]. Under the IDF definition (Table 1) [43], an individual is deemed to have MetS if they have central obesity (waist circumference ≥90 cm for South and East Asian men and ≥80 cm for South and East Asian women, with ethnicity-specific values, assumed if BMI is >30 kg/m^2^), plus any two of the following four factors: (1) raised triglycerides (≥150 mg/dL) or specific treatment for this lipid abnormality; (2) reduced high-density lipoprotein (HDL) cholesterol (<40 mg/dL in males, <50 mg/dL in females) or specific treatment for this lipid abnormality; (3) raised blood pressure (blood pressure ≥130/85 mm Hg) or treatment of previously identified hypertension; and (4) raised fasting plasma glucose (≥100 mg/dL) or previously diagnosed T2DM.

*Body mass index*. Body mass index (BMI) is a measure of body fat based on height and weight that applies to adult men and women. It is defined as body mass in kilograms (kg) divided by the square root of body height in meters (m), expressed in units of kg/m^2^. We used the Asian-specific cut-off points of the BMI for overweight and obesity (23.0 and 27.5 kg/m^2^) recommended by the World Health Organization (WHO) [44]. An individual with a BMI lower than 23 was considered underweight or normal weight, 23 to 27.4 as overweight, and 27.5 or higher as obese.

*Demographic characteristics*. We conducted the analysis separately by sex (man or woman). We also accounted for age (in years), marital status (currently married or not), education (high school or below, college or some college, or graduate degree), and poverty level (ratio of annual family income to federal poverty line).

*Modifiable lifestyle behaviors*. To quantify the energy expenditure of physical activity, we calculated the metabolic equivalent task (MET) scores by multiplying the frequency, duration, and NHANES-recommended metabolic-equivalent (MET) values (8.0 for vigorous and 4.0 for moderate intensity) [45]. We then developed four categories based on the cut-off points from the 2018 Physical Activity Guidelines for Americans [46]: sedentary (those reporting no regular physical activities), insufficient (those performing 1–499 MET-min of activities per week), moderate (those performing 500–1000 MET-min of activities per week), and high (those performing greater than 1000 MET-min of activities per week) [47]. Tobacco use was measured in two categories: whether an individual currently uses any tobacco products or not. In addition, we measured the amount and frequency of alcohol use in four categories [48]. Lifetime abstainers reported having consumed fewer than 12 drinks in their lifetime. Former drinkers reported over 12 drinks in their lifetime but none in the past year. Non-excessive current drinkers reported 14 or fewer drinks per week on average for men or 7 or fewer drinks per week on average for women. Excessive current drinkers reported more than 14 drinks per week on average for men or more than 7 drinks per week on average for women, or 5 or more drinks in a single day at least once in the past year for either sex.

### 2.3. Statistical Analysis

We applied the appropriate sample weights according to the National Center for Health Statistics guidelines to account for the complex survey design, the oversampling of Asian Americans, and taking into account that data on fasting glucose were collected on a subsample of the population [49]. We used the *svy* command in Stata to apply the weights. To handle missing values on laboratory and examination variables, we employed multiple imputation (MI), a commonly used model-based approach for dealing with missing data. Using this technique, we replaced each missing value with multiple imputed values to create multiple complete datasets. Each dataset was analyzed separately, and the results were combined to obtain valid statistical inferences [50]. A *p*-value of 0.05 or below was considered statistically significant. All data analyses were conducted in Stata 16 [51].

We computed age-adjusted, sex-specific MetS prevalence and each MetS component separately for each of the three BMI levels. Specifically, we used the age distribution in the US 2010 Census to standardize the prevalence and means of MetS and the five components. All age-adjusted estimated prevalence rates were presented as a percentage with 95% confidence intervals (CIs).

Descriptive statistics of BMI, five sociodemographic characteristics, and three modifiable lifestyle behaviors among the Asian American sample were presented, alongside with those of the reference group, the NHW sample. On top of that, we conducted multivariate binary logistic regression to examine the risks of MetS in Asian Americans in comparison to NHWs across the continuum of BMI. Specially, we regressed Asian American ethnicity (vs. NHW), BMI in both linear and quadratic terms (i.e., BMI and BMI squared), and an interaction between Asian American ethnicity and BMI in both terms, on MetS, while controlling for age, age squared, sex, marital status, education, poverty level, physical activity, alcohol use, and tobacco use. By including squared terms for BMI and age, we were able to model nonlinear associations between these covariates and MetS. Predicted probabilities and 95% CIs of MetS were computed for Asian Americans and NHWs separately at various levels of BMI (from 16 to 34) and separately for men and women.

## 3. Results

From NHANES, this study focused on the subsample of 2121 participants who were 18 years old or older and self-identified as non-Hispanic Asian. As a reference group, we used the subsample of 6318 NHW participants who were 18 years or older. The descriptive statistics of sociodemographic characteristics and modifiable lifestyle behaviors are presented in Table 2, separately for the two racial groups. The Asian American sample had an average age of 44.18, with 53.55% female and 46.55% male. The average/mean BMI was 24.77. Most of the Asian American participants were currently married (65.68%) and had college or higher degrees (51.55%). The average family income to federal poverty line ratio was 3.06. About two-thirds had not been physically active (24.20% sedentary and 44.73% insufficient). In addition, 9.30% were current tobacco users, 53.44% were non-excessive current drinkers, and 4.61% were excessive current drinkers. In comparison with the reference group, NHW subjects, the Asian American sample had a lower average age, a lower BMI, and a higher proportion of college degree holders. The Asian American sample had lower levels of current tobacco use and alcohol use, and a lower proportion of high/moderate level of physical activity.

Table 3 presents the age-adjusted, sex-specific prevalence of its five individual components (central obesity, raised triglycerides, reduced HDL, raised blood pressure, and raised fasting plasma glucose) by BMI levels among Asian Americans. We found that the estimated prevalence of MetS and all individual components increased with higher BMI levels in both sexes. Among Asian American men, the prevalence of MetS was 5.32% (95% CI: 2.36–8.29%) among men with <23 BMI, 38.23% (95% CI: 34.21–42.25%) in those with a BMI between 23 and 27.4, and 78.68% (95% CI: 72.30–83.07%) in those with a BMI 27.5 or higher. Similar trends were noted in Asian women, with 18.61% (95% CI: 14.36–22.85%) prevalence among women with <23 BMI, 47.82% (95% CI: 41.94–53.71%) in those with a BMI between 23 and 27.4, and 67.73% (95% CI: 59.91–75.55%) in those with a BMI 27.5 or higher. For the five individual components, we see the estimated rates increase from lower to higher BMI levels for both sexes.

**Figure 1:**
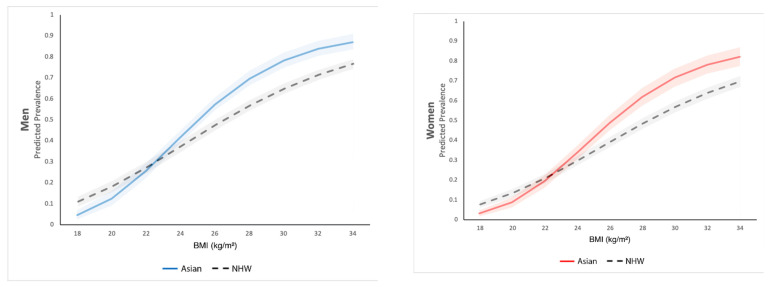
Predicted prevalence of MetS by BMI, compared between Asian Americans and non-Hispanic Whites.

We then conducted multivariate binary logistic regression on MetS and computed the predicted probabilities and 95% CIs of MetS for Asian Americans and NHWs separately at various levels of BMI (from 16 to 34), separately for men and women (Figure 1). Such results allow us to compare MetS risks for the two racial/ethnic groups along the BMI continuum, with sociodemographic and modifiable lifestyle behaviors held at the mean values. The predicted probabilities of MetS were lower in underweight or normal weight Asian American men than in their NHW counterparts with the same BMI, but the differences decreased as BMI went up, reaching zero at a BMI of approximately 22.65. As BMI increased further above 22.65, the predicted probabilities of MetS were higher in Asian American men than in NHW men with the same BMI. For example, the predicted probability of MetS was 0.78 for Asian American men with a 30 BMI, which was much higher than that of NHW men with the same or even greater BMI (0.65 for BMI 30, 0.71 for BMI 32, and 0.76 for BMI 34).

A similar trend was observed for the differences in MetS predicted probabilities between Asian American and NHW women. Asian American women with a BMI of 22.7+ were at greater risk for MetS than their NHW counterparts at the same BMI level, despite their lower propensity for MetS at lower BMI levels. The predicted probability of MetS was 0.72 for Asian American women with BMI 30, which was much higher than that of NHW women with the same or even greater BMI (0.57 for BMI 30, 0.64 for BMI 32, and 0.70 for BMI 34).

Our findings show that for both sexes, the predominance of MetS in Asian Americans start at the BMI level of approximately 22.7 and reach the highest at 30. The differences are statistically significant from approximately 24.5 and above for men and approximately 24.8 and above for women. With a BMI lower than 19.8 for men and 19.9 for women, Asian Americans have significantly lower probabilities of MetS than their NHW counterparts with the same BMI.

## 4. Discussion

The present study has three main findings. First, our results show that Asian Americans with a BMI above 23 are at elevated risk for MetS than NHWs with the same BMI, adding support to the use of BMI 23 as an Asian-specific cut-off point for ideal weight. This finding is not entirely consistent with previous findings. A study of 43,507 Asian American primary care patients over age 35 in the San Francisco Bay Area [20] found significantly higher MetS risk in Asian Americans than NHWs across all BMI levels and a higher risk for high blood pressure in Asian Americans than NHWs for those with a BMI level of 23 or above.

Though it remains unclear why Asian Americans have higher rates of metabolic disturbance at lower BMI levels compared to NHWs, one possible explanation relates to the differences in body fat content between ethnic groups. Previous studies have attributed the higher rates of MONW in Asians to greater abdominal fat accumulation [13]. Compared to NHWs with similar BMIs, Asians have 3–5% higher total body fat with some subgroups, with South Asians, specifically, being especially prone to developing abdominal obesity [52,53]. Furthermore, increases in weight are thought to be more detrimental in Asians than in other ethnic groups—every 11 pounds gained during adulthood leads to an 84% increased risk of T2DM [54]. This could also help to explain why Asian Americans with lower BMIs in our study appeared metabolically healthier than their NHW counterparts but became progressively more unhealthy with increasing BMI. Additionally, it has been hypothesized that certain Asian subgroups develop metabolic complications at a lower body weight due to having increased fat deposition in visceral rather than peripheral tissues, as well as exhausting their storage capacity of subcutaneous adipose tissue more quickly than NHWs [55]. Thus, research in this population could benefit from using supplemental clinical measures in addition to BMI as an index of measuring adiposity related to upper subcutaneous white adipose tissue, which is a strong predictor of MetS.

Our results paint a more nuanced relationship between BMI and MetS and the different patterns between the two racial/ethnic groups. Nonetheless, the finding of this study adds to the growing body of evidence that points to the metabolic risks in Asian Americans and, by extension, the need for behavioral and clinical interventions to prevent and manage cardiometabolic conditions in Asian Americans, especially those with a BMI of 23 or above. Community-based interventions that are culturally tailored for the needs of Asian American communities are needed to increase awareness of the cardiometabolic risks, to promote healthy lifestyle behaviors such as physical activity and stress management, and to facilitate community–clinic linkages to health care.

In addition, we found that while the prevalence of MetS and its individual components increases with higher BMI levels in both sexes, the relationship between body weight and cardiometabolic conditions is not as straightforward as it may seem. Just as not all obese individuals present with cardiovascular risk, not all lean individuals are metabolically healthy. Specifically, we found MetS rates of 5.32% and 18.61% among Asian American adult men and women with a BMI under 23, i.e., the metabolically obese but of normal body weight (MONW). Given that research has consistently found low awareness of hypertension and other cardiometabolic conditions among Asian Americans, it was possible that the awareness was even lower among MONW. This raises the question of whether Asian Americans who fall into the MONW category are aware of their metabolic conditions, and if so, whether they are taking measures to manage their conditions to prevent cardiovascular events. These questions remain unanswered and warrant future investigation.

Our findings also suggest that central obesity and low HDL cholesterol are more common in Asian American women, while high triglyceride levels, hypertension, and hyperglycemia were more widespread among Asian American men. This is likely due to sex-specific differences in pathophysiology of metabolic processes. For example, whereas premenopausal women tend to develop peripheral obesity with subcutaneous fat deposition, men are more likely to develop central obesity with visceral fat deposition. Visceral adipocytes are major sources of free fatty acids and cytokines that induce insulin resistance and atherosclerosis [56]. Thus, women require additional fat accumulation to achieve the same level of metabolic disturbance as men. Specific lifestyle factors, including physical exercise, also provide higher protective value in women [57]. Ultimately, such sex-specific differences suggest that the relative risk of MetS components varies in men versus women and that we must consider these differences when evaluating the risk of MetS and its associated cardiovascular events.

This study is not without limitations. First, while NHANES provides a large sample size for Asian Americans, there is likely an under sampling of undocumented immigrants [58], who may be more likely to be social economically underprivileged and less likely to have access to adequate preventive healthcare services. The MetS burden among this population warrants future research. Second, while we provided an estimate of MetS burden in Asian Americans, statistics are lacking for detailed Asian ethnic groups that are heterogeneous in socioeconomic standing and cardiovascular profile [19]. Research efforts are needed for ethnic-specific examination of the burden and epidemiological mechanisms of MetS, as well as identification of community and clinical solutions. Furthermore, we must acknowledge that the use of cross-sectional data limited our ability to draw causal inferences from our findings. Longitudinal research is needed to see how socio-behavioral factors influence the metabolic outcomes for Asian Americans in comparison to their NHW counterparts.

This is, to our best knowledge, the first study to provide an estimate of MetS prevalence in Asian Americans using a nationally representative sample. The study findings support the utilization of lower BMI ranges for defining overweight and obesity in Asian populations, which could allow for earlier and more appropriate screening for MetS within community and clinical settings. Our findings have significant research and clinical implications for better MetS prevention, diagnosis, and treatment in the Asian American community.

## Figures and Tables

**Table 1:** Metabolic syndrome as defined by the IDF criteria.

Criteria: Central Obesity (Defined as Waist Circumference ≥90 cm for South and East Asian Men and ≥80 cm for South and East Asian Women, with Ethnicity-Specific Values, Assumed if BMI is >30 kg/m^2^) Plus, Any Two of the Following Four Factors…	
Raised triglycerides	≥150 mg/dL
Reduced HDL cholesterol	Or specific treatment for this lipid abnormality
	<40 mg/dL in males; <50 mg/dL in females
	Or specific treatment for this lipid abnormality
Raised blood pressure	≥130/85 mmHg
	Or treatment of previously identified hypertension
Raised fasting plasma glucose	≥100 mg/dL
	Or previously diagnosed T2DM

**Table 2:** Descriptive statistics of BMI, sociodemographic characteristics and modifiable lifestyle behaviors for Asian American and non-Hispanic White samples.

	Asian Americans (*n* = 2121)	Non-Hispanic Whites (*n* = 6318)
Sociodemographic characteristics mean or % (se)		
BMI, mean	24.77 (0.11)	28.84 (0.14)
Age, mean	44.18 (0.69)	48.98 (0.43)
Family income to poverty line ratio (mean)	3.06 (0.08)	3.23 (0.07)
Sex		
Female	53.55% (0.01)	51.35% (0.01)
Male	46.45% (0.01)	48.65% (0.01)
Marital Status		
Currently married	65.68% (0.02)	56.73% (0.01)
Other	34.32% (0.02)	43.27% (0.01)
Educational Attainment		
<High school	13.85% (0.01)	9.39% (0.01)
High school or some college	34.60% (0.02)	55.42% (0.01)
≥College	51.55% (0.02)	35.19% (0.02)
Modifiable lifestyle behaviors mean or % (se)		
Physical Activity		
Sedentary	24.20% (0.01)	21.29% (0.01)
Insufficient	44.73% (0.45)	34.62% (0.01)
Moderate	15.75% (0.16)	17.00% (0.01)
High	15.12% (0.15)	27.08% (0.01)
Tobacco Use		
Current user	9.30% (0.01)	19.33% (0.01)
Former or never user	90.70% (0.01)	80.67% (0.01)
Alcohol Use		
Lifetime abstainer	31.11% (0.01)	9.68% (0.01)
Former drinker	10.84% (0.01)	13.81% (0.01)
Non-excessive current drinker	53.44% (0.02)	66.77% (0.02)
Excessive current drinker	4.61% (0.01)	9.75% (0.01)

**Table 3:** Age-adjusted, sex-specific prevalence of MetS and individual components by BMI level in Asian Americans, NHANES 2011–2016 (*n* = 2121).

	Men, % (95% CI)			Women, % (95% CI)		
BMI	<23 (under or Normal Weight)	23–27.4 (Overweight)	≥27.5 (Obese)	<23 (under or Normal Weight)	23–27.4 (Overweight)	≥ 27.5 (Obese)
MetS	5.32 (2.36–8.29)	38.23 (34.21–42.25)	77.68 (72.30–83.07)	18.61 (14.36–22.85)	47.82 (41.94–53.71)	67.73 (59.91–75.55)
Central obesity	6.78 (3.66–9.91)	53.99 (49.49–58.50)	97.82 (95.98–99.66)	35.93 (31.00–40.87)	88.55 (84.52–92.57)	99.96 (n/a)
Raised triglycerides	32.85 (26.47–39.24)	56.96 (53.50–60.42)	70.35 (64.46–76.24)	27.82 (23.56–32.08)	48.22 (42.69–53.75)	57.24 (49.75–64.74)
Reduced HDL cholesterol	19.72 (14.56–24.88)	39.27 (35.37–43.17)	55.12 (48.61–61.63)	27.26 (22.96–31.56)	41.22 (36.18–46.27)	59.46 (50.48–68.44)
Raised blood pressure	31.76 (26.01–37.51)	35.85 (31.39–40.31)	56.53 (50.53–62.52)	30.02 (26.11–33.94)	33.94 (29.28–38.61)	40.73 (35.18–46.28)
Raised fasting plasma glucose	49.84 (42.44–57.23)	59.88 (54.19–65.56)	72.40 (65.24–79.56)	33.56 (27.17–39.95)	41.94 (34.30–49.58)	60.57 (51.64–69.51)

## Data Availability

The datasets analyzed in this study, NHANES 2011–2016, are available on the website of the National Center for Health Statistics, https://wwwn.cdc.gov/nchs/nhanes/Default.aspx, accessed on 26 October 2018.
